# Intensive Interdisciplinary Rehabilitation in the Pediatric Hematology/Oncology Setting: Feasibility and Perceived Benefit of the Acute Neurological Injury Service

**DOI:** 10.3390/cancers16172999

**Published:** 2024-08-29

**Authors:** Darcy Raches, Amar Gajjar, Giles W. Robinson, Jason M. Ashford, Martina Bryndziar, April Huggins, Sherry Lockett, Allison Harris, Hannah Taylor, Ellen Bursi, Heather M. Conklin

**Affiliations:** 1 Department of Psychology and Biobehavioral Sciences, St. Jude Children’s Research Hospital, 262 Danny Thomas Place, Memphis, TN 38105, USA; 2 Division of Neuro-Oncology, St. Jude Children’s Research Hospital, 262 Danny Thomas Place, Memphis, TN 38105, USA; 3 Department Rehabilitation Services, St. Jude Children’s Research Hospital, 262 Danny Thomas Place, Memphis, TN 38105, USA

**Keywords:** pediatric hematology/oncology, interdisciplinary rehabilitation, posterior fossa syndrome, cerebellar mutism syndrome

## Abstract

**Simple Summary:**

Interdisciplinary rehabilitation more effectively promotes recovery from acquired brain injury than a single discipline approach. Little is known about the desirability and benefit of this approach for pediatric cancer patients. We show that an interdisciplinary approach within a specialized pediatric hematology/oncology hospital is manageable for caregivers of children with new cancer diagnoses. Parents found this approach helpful, including coordinated care planning, setting an interdisciplinary goal, parent brain injury education, cognitive assessment reports, and weekly cognitive intervention sessions. Parents wanted to have a peer mentor while managing new cancer diagnoses and later serve as a mentor for a newly diagnosed family. Other benefits of this approach should be explored.

**Abstract:**

(1) Background: Intensive interdisciplinary rehabilitation services more effectively promote recovery from acquired brain injury than a single discipline approach. However, research literature is lacking regarding the perceived feasibility and utility of an interdisciplinary approach across disciplines for patients within a tertiary care pediatric hematology/oncology setting. (2) Methods: The Acute Neurological Injury (ANI) service applied an acquired brain injury/inpatient rehabilitation interdisciplinary approach to a pediatric hematology/oncology population, with a focus on interdisciplinary communication, shared goal setting, and coordinated transition planning. Caregivers whose children received coordinated ANI program care were interviewed regarding the perceived feasibility and utility of ANI program components. (3) Results: An interdisciplinary approach to a pediatric hematology/oncology population is feasible for caregivers and for providers of rehabilitation and psychosocial services within a tertiary care cancer hospital setting. Parents perceived benefits from aspects of this approach including coordinated interdisciplinary care planning, the implementation of an interdisciplinary goal, parent brain injury education, neuropsychological assessment reports, and weekly cognitive intervention sessions. Parents were interested in both having a peer mentor while managing new cancer diagnoses and later serving in a mentor role for a newly diagnosed family. (4) Conclusions: An interdisciplinary acquired brain injury approach to a pediatric hematology/oncology population is feasible with perceived benefits to families managing new cancer diagnoses.

## 1. Introduction

Intensive interdisciplinary rehabilitation services, such as those provided in inpatient rehabilitation settings, are more effective in promoting recovery from acquired brain injury than a single discipline approach [1,2,3]. Greater functional gains for patients are associated with collaboration among treating professionals (i.e., nursing, physical therapy [PT], occupational therapy [OT], speech/language therapy [SLP], clinical psychology, neuropsychology, rehabilitation medicine, counseling, and social work) [4] in communication [5], goal-setting, and decision-making [6], which is facilitated by meeting on a regular basis [1]. Some institutions have developed oncology rehabilitation programs specifically designed to meet the unique needs of a pediatric oncology population. These models, however, have either focused on a narrow age range including only very young children [7,8] or have focused on an interdisciplinary approach only amongst rehabilitation providers despite acknowledging that the inclusion of providers outside of the rehabilitation realm would likely be beneficial [9,10,11]. Thus, there is no research literature o date regarding the perceived feasibility and utility of an interdisciplinary approach across disciplines for a wide range of patients within a tertiary care pediatric hematology/oncology setting.

Children with a new cancer diagnosis are at risk for experiencing adverse neurological sequelae given central nervous system (CNS) complications such as infection, stroke, methotrexate neurotoxicity, and posterior reversible encephalopathy syndrome (PRES) are more common in children receiving cancer-directed treatment [12,13,14,15]. Brain tumor patients have additional neurological risk including focal neurological deficits due to the mass effect of a brain tumor and associated biopsy/resection. Up to 29% of children who undergo surgical resection of posterior fossa brain tumors experience postoperative posterior fossa syndrome (PFS)/cerebellar mutism syndrome (CMS) characterized by speech/language changes, motor impairments, and emotional lability [16,17,18]. While deficits associated with PFS/CMS improve in the days and weeks following resection, many impairments persist [19,20,21,22,23]. Further, indwelling CNS devices, such as VP shunts, increase the risk of CNS infection in brain tumor patients [24,25]. There is also a risk for neurological injury related to adjuvant brain tumor treatment, such as radiation necrosis occurring in approximately 5% of pediatric brain tumor patients [26,27].

Given the above-mentioned high incidence of cognitive and physical deficits in children with a new cancer diagnosis, this population would benefit from an intensive interdisciplinary rehabilitation approach. However, due to the need to initiate cancer treatment promptly, inpatient rehabilitation is often delayed for pediatric oncology patients, or forgone altogether. Historically, patients with needs across disciplines were receiving siloed intervention at our institution to address isolated needs within each area of functioning. This was true despite the medical complexity or severity of the needs of each patient. As a result, opportunities were missed for each member of the treatment team to contribute to the understanding of other team members to facilitate a comprehensive understanding of patient needs. To address these competing treatment needs, we created the Acute Neurological Injury (ANI) service. We drew from the acquired brain injury/inpatient rehabilitation literature and applied a similar interdisciplinary approach to a pediatric hematology/oncology population, with a focus on interdisciplinary communication, shared goal setting, and coordinated transition planning. The overarching aim for this study was to identify the feasibility and perceived benefit of the ANI service to better address acute rehabilitation needs within a specialized tertiary pediatric hematology/oncology treatment center setting.

## 2. Materials and Methods

### 2.1. Setting

St Jude Children’s Research Hospital is a nationally renowned private, non-profit, pediatric academic medical center with a mission of advancing cures for pediatric catastrophic diseases through research and treatment, with an emphasis on hematological and oncological diagnoses. The hospital includes outpatient clinics and more than 70 inpatient beds. Treatment, travel, housing, and food are provided irrespective of ability to pay to patient families throughout a child’s care.

### 2.2. ANI Service

#### 2.2.1. ANI Service Development

The ANI service was initiated in 2016 to optimize outcomes for patients with new onset neurological insult that resulted in cognitive changes and functional deficits requiring intervention from all rehabilitation disciplines (i.e., SLP, OT, and PT). Based on an inpatient rehabilitation approach, the ANI team consists of members from the departments of psychology (i.e., pediatric psychology and neuropsychology), rehabilitation services (i.e., SLP, OT, inpatient and outpatient PT, and a rehabilitation coordinator), nursing, child life (i.e., a trained professional who provides tailored information, support, and guidance to pediatric patients and family members regarding medical diagnoses, procedures, and the medical setting), social work, and the hospital school program. Patients are identified for inclusion in the ANI service by primary treating physicians as well as ANI team members and other clinicians within represented disciplines. Patients are seen by individual services for evaluation and intervention on a schedule that is determined by each provider based on each patient’s clinical needs. Given the significant rehabilitation needs of the patients on the ANI service, these patients are scheduled for more frequent rehabilitation sessions than peers with similar hematology/oncology diagnoses. Neuropsychology follows patients on the ANI service throughout their active cancer treatment with serial assessment at important transitions between components of cancer treatment (e.g., after a surgical resection, and before and after radiation and/or chemotherapy). Initial parent education is provided about brain behavior relationships specific to each child’s diagnosis, and weekly neuropsychological cognitive remediation sessions are scheduled to address weaknesses identified via serial assessments.

#### 2.2.2. Interdisciplinary Communication

Interdisciplinary communication is facilitated through bimonthly ANI meetings. A patient list is circulated via email to ANI team members in the days before each meeting, which includes a concise medical summary and recent updates for each listed patient. The ANI team member from each discipline gathers information from other providers within their discipline for presentation at each ANI meeting. For example, the ANI team OT would gather information from other OT providers working with any patient on the ANI list in advance of each ANI meeting and would present pertinent information within the ANI meeting about those patients. This efficient approach to meeting preparation allows for all active patients being followed (n = 3–13) to be discussed in each hour-long ANI meeting. Updates, recent events, and concerns are discussed for each patient as well as future planning. In this way, problems may be identified early before there is opportunity for issues to become exacerbated, and concerns may be consistently addressed across all disciplines. A meeting summary document is then circulated via email after each meeting for each ANI team member to report back to other providers within their discipline. Trainees within each discipline are welcome to participate in this interdisciplinary training opportunity.

This interdisciplinary approach also allows for the cross-education of team members regarding discipline specific expertise, which ultimately deepens each provider’s understanding of the ANI service patients. For example, neuropsychologists might highlight brain–behavior relationships contributing to current presentations, psychologists may share behavior management approaches to enhance compliance, speech language pathologists may provide recommendations to optimize methods of communication, physical therapists may provide guidance surrounding positioning and mobility needs, and occupational therapists might identify accommodations to improve pointing accuracy relevant to assessment and communication.

#### 2.2.3. Shared Goal Setting

An interdisciplinary goal is established for each patient followed by the ANI service so all providers working with each of these patients are contributing to progress in an area of identified need. It is expected that the identified goal will be implemented in each session for all providers whose disciplines are represented within the ANI clinic. As such, interdisciplinary goals allow for increased practice of a particular skill across different settings and with different providers to both improve generalizability and provide multiple sources of information about success with and/or barriers to each goal. The content of each goal is determined based on each individual patient’s needs and each goal is agreed upon by all ANI team members. Interdisciplinary goals that would have practical implications across disciplines are often prioritized. For example, the increasing use of a hemiparetic arm may help a patient make gains with positioning in PT, activities of daily living in OT, handwriting in school, and completing tasks with manipulatives in language therapy. Goals to target an area in which the patient has made slow gains or areas in which patients and families have voiced specific interest are often prioritized (e.g., safe transfers).

Progress on interdisciplinary goals is reviewed at bimonthly meetings and goals are updated or changed regularly to optimize progress for these high-need patients. Interdisciplinary goals are constructed in a S.M.A.R.T. framework (i.e., specific, measurable, achievable, relevant, and time-bound) to optimize the effectiveness of each goal and to assure that it is easy to determine whether each patient has met their interdisciplinary goal within recent weeks. Goals may target rehabilitation needs such as motor deficits (e.g., the patient will use her weaker right hand three times in each session) or communication (e.g., the patient will direct visual gaze to a named item from a field of 2, on 3 occasions each session). Cognitive skills such as orientation and memory are often the target of interdisciplinary goals (e.g., the patient will recall his medical record number one time within each session when this is reviewed at the start of session) as are behavior or difficulties with adherence (e.g., the patient will be compliant with tasks on the first request 50% of the time with the use of a visual schedule).

#### 2.2.4. Coordinated Transition Planning

Needs are identified and collaboratively addressed in advance of transition times, such as facilitating the establishment of rehabilitation services during brief times at home between aspects of cancer-directed treatment and the transition home at the end of treatment. End of treatment evaluations are coordinated. The rehabilitation coordinator works closely with social work to identify needs and ensure barriers to necessary services are addressed prior to transitioning home. The rehabilitation coordinator further ensures rehabilitation providers within the home community have the necessary documentation to facilitate continuity of care upon return home. Neuropsychology and hospital school personnel help with academic planning (e.g., helping to establish an individualized education program [IEP] or section 504 plan as appropriate). Child life is available to provide education about a returning patient’s diagnosis to his or her schoolmates as desired.

### 2.3. Caregiver Interview

Caregivers of children and young adults who received coordinated care as part of the ANI program were interviewed regarding the perceived feasibility and utility of ANI program components including parent psychoeducation, neuropsychological assessment, cognitive remediation, interdisciplinary team coordination/goal setting, and parental supports. To assure homogeneity in the type and duration of adjuvant cancer-directed treatment received, caregiver interviews were targeted to the most frequent diagnosis and reason for referral to the ANI service. Thus, only caregivers of patients with PFS after medulloblastoma resection who received cranial-spinal irradiation (CSI) followed by chemotherapy per a multi-institutional treatment protocol were approached for interview. Dichotomous yes/no responses were gathered as well as responses regarding the perceived utility of aspects of the interdisciplinary ANI program approach via a five-point Likert scale. Additional open-ended questions were posed to caregivers to collect additional information. All interviews were completed at least 4 months following the completion of cancer-directed treatment.

## 3. Results

### 3.1. ANI Service Participants

Within the first six years of the ANI service (2016–2022), 100 patients have been followed, with a larger percentage of males represented in this group (56%) (Table 1). Patients ranged in age from 2 to 21 years (mean 8 years, SD = 4.86). Ten percent received their cancer diagnosis and initial treatment (i.e., brain tumor resection) outside of the United States (US) and later came to our hospital for adjuvant treatment, while the remainder of patients received all treatment within the continental US. Patients residing within the US were an average of 577 miles from home while receiving treatment at St. Jude (range 11–2017 miles).

The majority of the patients followed by the ANI service had a brain tumor diagnosis (95%), with the remainder having a diagnosis of leukemia/lymphoma (3%), solid tumor (1%), or hemophagocytic lymphohistiocytosis (1%). Most of the referred brain tumor patients had a diagnosis of medulloblastoma (74%), followed by ependymoma (6%), atypical teratoid rhabdoid tumor (4%), and diffuse intrinsic pontine glioma (2%). The most common reason for referral to the ANI service was PFS/CMS (81%), followed by mental status changes (10%) and stroke (4%), with less frequent radiation necrosis, encephalitis, meningitis, prolonged extracorporeal membrane oxygenation, or memory deficits (1% each). These demographics reflect that the ANI service initially targeted the needs of patients experiencing PFS/CMS and later expanded to address the acute needs of other patient populations. Patients were followed within the ANI service for the duration of their active treatment (i.e., 1.5–9 months) unless cognitive concerns resolved and/or rehabilitation services were deemed no longer necessary.

### 3.2. Caregiver Interview Participants

Families were considered for interview if their child experienced PFS/CMS following medulloblastoma resection and they received adjuvant cancer-directed treatment per a multi-institutional treatment protocol (n = 68). Families were not approached for interview if they were bereaved (n = 4), international families of children for whom English was not the primary language (n = 5), lost to hospital follow-up (n = 2), or if they had only a single contact with neuropsychology (n = 8). Forty-nine families were identified for participation in the caregiver interview, and thirty-six (73%) of those families agreed to participate. All interviewees were parents, and they were interviewed by a research assistant who was not a member of the ANI team. The majority of patients discussed were male (61%) with a mean age of 9 years (SD 4.9). Most underwent a single surgical tumor resection (86%) and several required CSF diversion (42%). All received adjuvant cancer-directed treatment including CSI with focal boosts to tumor sites followed by either four courses (25%) or seven courses (75%) of chemotherapy. Seventy-five percent (n = 27) experienced PFS/CMS with a period of frank mutism while the remainder experienced PFS/CMS with reduced speech only.

### 3.3. Caregiver-Perceived ANI Feasibility and Benefit

**Figure 1:**
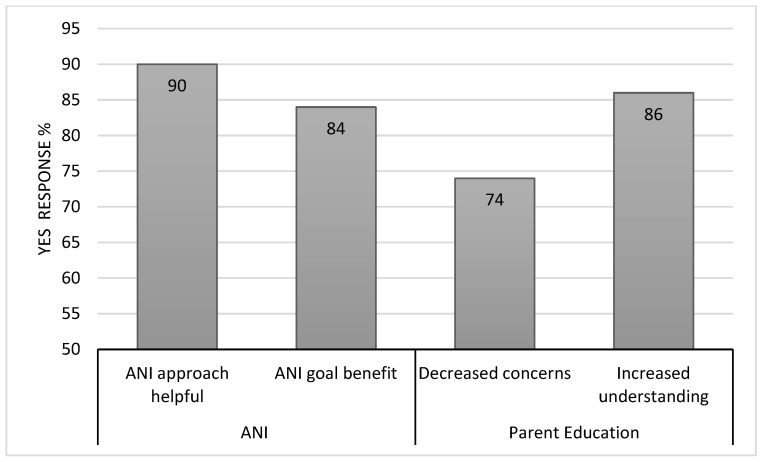
Perceived ANI benefit.

**Figure 2:**
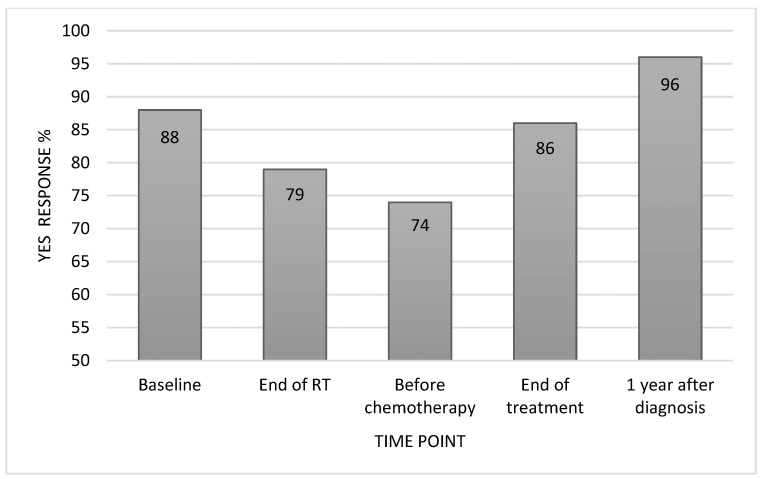
Neuropsychological assessment benefit by timepoint.

**Figure 3:**
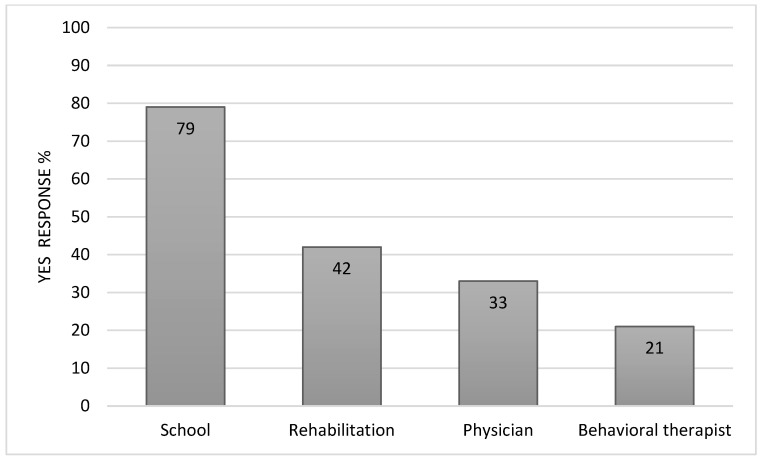
Neuropsychological assessment shared.

Many parents reported that initial psychoeducation about PFS decreased their concerns (74%) and increased their understanding of their child in the context of PFS (86%) (Figure 1). They reported benefit from neuropsychological assessment reports prior to adjuvant cancer-directed treatment (88%), at the end of treatment (86%), and at 1 year following the initiation of cancer treatment (96%) (Figure 2). They perceived less benefit from assessments intended to inform provider interventions during treatment (74% and 79%). Reports were shared most often with schools (79%), rehabilitation specialists (42%), physicians (33%), and behavioral therapists (21%) (Figure 3). It was frequently noted that the recommendations to assist with implementing school accommodations were the most helpful part of the neuropsychological assessment reports. Parents also reported that they referred to the neuropsychological reports themselves (66%), most often reviewing the recommendations from the end of treatment report and the evaluation completed at 1 year following the initiation of cancer treatment. Some patients (n = 11) were referred by their primary oncologist for additional neuropsychological assessment at a later timepoint (e.g., when transitioning to high school, when turning 18, or five years after the end of treatment). All of these families found the additional neuropsychological assessment to be helpful (100%).

Parents reported that the interdisciplinary ANI program approach was helpful (90%), and the coordinated interdisciplinary goal was beneficial (84%) (Figure 1). They noted that the interdisciplinary approach is “critical” or “invaluable”, adding that “it takes the burden away from families as parents don’t have to communicate everything between providers”. Some lamented that this coordinated approach did not continue once they returned to the home community, noting that “No one is on the same page at home; providers don’t interact”, though there was also mention that the interdisciplinary ANI approach employed during adjuvant cancer-directed treatment “seemed secondary to the main goal of beating disease”.

**Figure 4:**
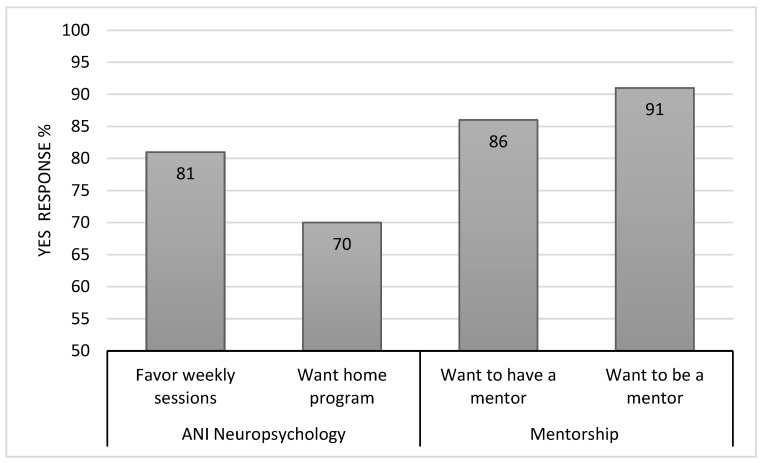
ANI feasibility.

Caregivers favored the weekly frequency of cognitive remediation sessions (81%), though fewer were interested in being provided additional cognitive remediation activities to work on at home between sessions (70%) (Figure 4). Many caregivers were interested in a formal peer support mentoring program. Parents were interested in both receiving a peer mentor to help them through the initial stages of managing new medulloblastoma and PFS/CMS diagnoses (86%), and later serving in a mentor role for a newly diagnosed family (91%) (Figure 4).

## 4. Discussion

We demonstrated that an acquired brain injury/inpatient rehabilitation interdisciplinary approach to the care of neurologically complex pediatric cancer patients with a focus on interdisciplinary communication, shared goal setting, and coordinated transition planning can be implemented and sustained within a tertiary care pediatric hematology/oncology setting. Further, this approach is perceived to be feasible by caregivers of children who are managing both a new brain tumor diagnosis and associated active cancer treatment. This was the case despite the complex postoperative course experienced by the patients whose families we interviewed as their children were affected by PFS/CMS, and the intensive adjuvant cancer-directed treatment they received including several weeks of radiation therapy as well as several months of chemotherapy. It is reasonable to consider that families whose children had a less complex postoperative course and/or less intensive treatment demands may find the additional appointments associated with the ANI service even more feasible.

Caregiver-perceived benefit from the interdisciplinary communication and shared goal setting of the interdisciplinary ANI approach is notable in our setting since both the medical team and families are often primarily focused on the lifesaving cancer treatment that patients are receiving. This is in contrast to an inpatient rehabilitation setting where the main focus is on the rehabilitation efforts.

The desire by families affected by PFS/CMS to receive information about this syndrome in the postoperative period has been documented [28]. We were encouraged to receive confirmation that caregivers within our cohort who received this kind of parent education identified this as being helpful. While it is not surprising that having this information only modestly decreased caregivers’ concerns about their child’s PFS/CMS diagnosis, it is reassuring that the majority of caregivers found that having this information helped them to better understand their child in the acute stages of PFS/CMS. Taken together, this suggests that education about brain behavior relationships has great potential to benefit families whose children have experienced a neurological complication associated with their child’s cancer diagnosis and associated treatment.

Serial cognitive screening throughout the course of a child’s active cancer treatment is not traditionally a component of care provided by neuropsychology. However, this was incorporated as a standard element of the ANI service given the acute neurological injury experienced by those followed by this service and the trajectory of recovery that children are likely to display in the weeks and months following this type of injury. There was a clear parent-perceived benefit from evaluations completed at the initiation and end of treatment. Additional interview responses suggest that baseline assessments contributed to the parents’ understanding of their child in the context of a recent PFS/CMS diagnosis while end-of-treatment assessments were beneficial in helping to establish services within the school setting upon the transition home. There was overwhelming benefit reported by nearly all families interviewed from the assessment completed one year following their child’s brain tumor diagnosis. Of note, the evaluation at this timepoint more closely resembled a traditional comprehensive neuropsychological assessment with the inclusion of standardized, age-referenced assessment measures. Again, the role of this assessment in helping children receive appropriate accommodations and other services within the school setting was frequently cited when describing why this assessment was felt to be beneficial. This is consistent with reports that families most often shared neuropsychological assessment reports with schools. Taken together, the results suggest targeted neuropsychological evaluations are warranted for oncology patients whose course is complicated by an acute neurological injury, such as PFS/CMS. Targeting the initiation and end of treatment with this type of assessment is advised, as well as completing a more comprehensive assessment 3–6 months following the end of cancer-directed treatment.

It is uncertain what contributed to the evaluations competed during active therapy being perceived as helpful by fewer caregivers. Some caregivers did not have automatic access to the reports from these interim evaluations unless they were requested from the hospital medical records department since those reports were intended to inform internal provision of care within the hospital. Although these reports were available to parents, directly or indirectly, they were not encouraged to share them with other providers since the internal providers had direct access to the report content. Thus, a limited awareness of these interim reports may have contributed to their being perceived as less helpful than other assessment reports.

The high percentage of parents who voiced interest in peer mentorship was notable. Caregivers shared a desire to have an experienced parent available to aid them while they managed the initial stages of adjusting to a new brain tumor and PFS/CMS diagnosis. Additionally, many parents also shared a desire to serve in that type of mentorship role for a less experienced family. Given this robust response to these mentorship questions we have leveraged the existing parent mentor program at our institution [29] and have formed a PFS/CMS parent mentor cohort. Thus, this type of support is now available to families whose children are newly diagnosed with a brain tumor and PFS/CMS.

### Limitations and Future Directions:

The exclusion of families who only had one interaction with neuropsychology may have resulted in an overestimate of the feasibility of participating in the ANI service as it is possible that the families who attended fewer sessions may have done so because they found attending the additional ANI sessions overwhelming.

Limiting the present study to only those patients with both recent medulloblastoma and PFS/CMS diagnoses may have also influenced the findings of this survey. Interviewing other patient groups who participated in the ANI service will be beneficial to determine if similar benefits are perceived by families managing other acute neurologic injuries during their active cancer treatment.

While the parent-perceived feasibility and benefit of the ANI service are clear from the present study, the financial cost/benefit ratio of this type of coordinated approach to care within a tertiary care pediatric hematology/oncology setting is unknown. It will be important to demonstrate added benefit and/or improved outcomes with this interdisciplinary approach as compared to the prior, siloed, approach in terms of continuity of care (e.g., successful transition to rehabilitation providers within the community, and the establishment of formal academic accommodations and related services). An exploration of the financial cost/benefit ratio of this approach should also consider factors that may influence how effectively this model could be exported to institutions with fewer resources. Of note, one of the main additional resources when comparing the ANI approach to the traditional siloed approach is the ANI interdisciplinary meeting, since most cancer centers offer the individual rehabilitation services with providers engaging in separate goal setting. The ANI approach limits this time burden by having a discipline representative and streamlined communication via structured patient tracking and emails. Perhaps the biggest increase in resources with the ANI approach may be the increased frequency in neuropsychological assessment. Parents perceived benefit from this component of the ANI approach, though the frequency of assessment could potentially be reduced to target the baseline evaluation timepoint and coordinated transition planning, which parents perceived greatest benefit from. This is consistent with the literature supporting the benefit of coordinated transition planning for an acquired brain injury population [30]. Future work to directly address these questions is warranted.

Future research may also inspect the perceived benefit to internal providers of the ANI service. Hospital clinicians may find additional benefit in the program components that families perceived to be beneficial. However, there is also potential that internal providers may also find other program components beneficial to optimizing patient care, such as the neuropsychological evaluations completed during patients’ active cancer treatment.

There is additional opportunity to leverage the coordinated ANI approach to assessment when transitioning between aspects of cancer-directed treatment (e.g., before and after radiation and/or chemotherapy) in order to learn more about this acutely impaired patient population. The degree of impairment experienced by these high-need patients early in their cancer-directed care often results in them being omitted from formal baseline assessments that provide valuable information to each individual patient. They are additionally all too often omitted from the research literature, both case series and broader prospectively designed studies, for similar reasons [21]. The acute assessment component of this ANI approach can thus help to fill this important gap in the literature [31].

## 5. Conclusions

We provide evidence that an acquired brain injury/inpatient rehabilitation interdisciplinary approach to a pediatric hematology/oncology population is feasible for providers of rehabilitation and psychosocial services within a tertiary care cancer hospital setting. This approach is similarly feasible with perceived benefits to families managing new PFS/CMS and medulloblastoma diagnoses and receiving cancer treatment, despite the multiple demands placed on caregivers during the stressful time surrounding their child’s cancer diagnosis and associated treatment.

Caregivers perceived benefit from neuropsychological assessment reports that were intended to be shared with providers outside of our hospital setting, such as school personnel and rehabilitation professionals within their home community. Weekly cognitive intervention sessions were preferred though fewer caregivers were interested in a home program. Caregivers voiced interest in both having a mentor to guide them through the active treatment process and later serving as a mentor to families with a child on active treatment.

The current findings suggest that an interdisciplinary rehabilitation approach, which has well-documented benefits for a pediatric acquired brain injury population, is feasible and has many similar benefits when applied to a neurologically complicated pediatric hematology/oncology population.

## Figures and Tables

**Table 1:** ANI service demographics (n = 100).

Sex			
Male (%)			56
Female			44
Age at diagnosis (years)			
Mean (SD)			8.84 (4.86)
Range			2–21
Distance from hospital (miles)			
Mean (SD)			577 (417)
Range			11–2017
Cancer diagnosis (%)			
	Brain tumor		95
		Medulloblastoma	74
		Ependymoma	6
		Atypical teratoid rhabdoid tumor	4
		Diffuse intrinsic pontine glioma	2
		Other *	9
	Leukemia		2
	Lymphoma		1
	Hemophagocytic lymphohistiocytosis		1
	Neuroblastoma		1
ANI referral reason (%)			
	Posterior fossa/cerebellar mutism syndrome		81
	Mental status change		10
	Stroke		4
	Other **		5

* Other brain tumors: non-germinomatous germ cell tumor, cerebellar juvenile pilocytic astrocytoma, embryonal tumor with multilayered rosettes, craniopharyngioma, ganglioglioma, germinoma, glioblastoma, glioma, or pineoblastoma. ** Other ANI referral reasons: radiation necrosis, encephalitis, meticillin-sensitive staphylococcus. aureus meningitis, prolonged extracorporeal membrane oxygenation, or memory deficits.

## Data Availability

Data will be made available upon reasonable request via de-identified data sets shared through HIPAA-compliant and secured data transfer programs.
